# Supplementary material for: Real-time reverse transcription polymerase chain reaction development for rapid detection of Tomato brown rugose fruit virus and comparison with other techniques
Source: PeerJ. 2019 Oct 17;7:e7928. doi: 10.7717/peerj.7928 (PMC6800982; doi:10.7717/peerj.7928)
Supplement: Supplemental Information 1 [file peerj-07-7928-s001.docx]

>7R-3*

GTAAGGCTTGCAAAATTTCGTTCGAAGACCGGAAAAAAGTTTAGTAGTAAAAGTGAGAATAATAGTGGTAATAATAGGCCGAAACCAGACAAAAACCAAAG

>7R-4*

GTAAGGCTTGCAAAATTTCGTTCGAAGACCGGAAAAAAGTTTAGTAGTAAAAGTGAGAATAATAGTGGTAATAATAGGCCGAAACCAGACAAAAACCAAAG

>8R-3*

GTAAGGCTTGCAAAATTTCGTTCGAAGACCGGAAAAAAGTTTAGTAGTAAAAGTGAGAATAATAGTGGTAATAATAGGCCGAAACCAGACAAAAACCAAAG

>8R-4*

GTAAGGCTTGCAAAATTTCGTTCGAAGACCGGAAAAAAGTTTAGTAGTAAAAGTGAGAATAATAGTGGTAATAATAGGCCGAAACCAGACAAAAACCAAAG

>9R-3*

GTAAGGCTTGCAAAATTTCGTTCGAAGACCGGAAAAAAGTTTAGTAGTAAAAGTGAGAATAATAGTGGTAATAATAGGCCGAAACCAGACAAAAACCAAAG

>9R-4*

GTAAGGCTTGCAAAATTTCGTTCGAAGACCGGAAAAAAGTTTAGTAGTAAAAGTGAGAATAATAGTGGTAATAATAGGCCGAAACCAGACAAAAACCAAAG
