# Supplementary material for: Real-time reverse transcription polymerase chain reaction development for rapid detection of Tomato brown rugose fruit virus and comparison with other techniques
Source: PeerJ. 2019 Oct 17;7:e7928. doi: 10.7717/peerj.7928 (PMC6800982; doi:10.7717/peerj.7928)
Supplement: Supplemental Information 3 [file peerj-07-7928-s003.docx]

| **Virus name** | **No. full genome analyzed** | **ToB5520F** | | **ToB-probe5558** | | **ToB5598R** | | **Acc. No. with the highest homology sequences inside the species** |
| --- | --- | --- | --- | --- | --- | --- | --- | --- |
|  |  | **position** | **Hybridisation %** | **position** | **Hybridisation %** | **position** | **Hybridisation %** |  |
| *Bell pepper mottle virus* | 1 | 5492 | 79.2 | 3120 | 68.2 | 23 | 65.2 | DQ355023 |
| *Brugmansia mild mottle virus* | 1 | 4216 | 65.2 | 5599 | 63.6 | 5360 | 65.2 | AM398436 |
| *Obuda pepper virus* | 2 | 2196 | 62.5 | 2815 | 68.2 | 4893 | 65.2 | D13438  L11665 |
| *Paprika mild mottle virus* | 2 | 392 | 66.7 | 3225 | 63.6 | 3206 | 60.9 | KX187305  AB089381 |
| *Pepper mild mottle virus* | 13 | 5306 | 66.7 | 4005 | 63.6 | 3560 | 69.6 | KP345899 - MH063882 - KR108207 -KR108206 - MG515725 - AB069853 - KU312319 - LC082100 - LC082099 - AB550911 - AB126003 - AB000709 -  AJ308228 |
| *Rehmannia mosaic virus* | 6 | 5518 | 75 | 6094 | 68.2 | 6283 | 65.2 | KU133476 - EF375551 - MF348202 - JX575184 - AB628188 - MG418836 |
| *Tobacco mild green mosaic virus* | 7 | 2584 | 66.7 | 3556 | 63.6 | 2810 | 65.2 | M34077 - KM596785 - MH730970 - DQ821941 - JX534224 - EF469769 -  AB078435 |
| *Tobacco mosaic virus* | 10 | 5515 | 79.2 | 6094 | 72.7 | 6283 | 60.9 | AF395129 - AF395128 - AF395127 -MK087763 - MG516107 - AF165190 -KY810785 - KF972436 - KF972435 - KF972434 |
| ***Tomato brown rugose fruit virus*** | 5 | 5520 | **100** | 5556 | **100** | 5598 | **100** | KX619418 - MK133095 - MK133093 - KT383474 - MN167466 |
| *Tomato mosaic virus* | 10 | 5518 | 70.8 | 3863 | 63.6 | 5733 | 60.9 | KR537870 - AF155507 - KX711903 - X02144 -AF332868 - AJ417701 -  MH507166 - MH507165 - MG456601 - KY912162 |
| *Tomato mottle mosaic virus* | 5 | 5522 | 79.2 | 4913 | 63.6 | 3919 | 65.2 | MH128145 - KR824951 - KR824950 - KT810183 - KP202857 |
